# Is *Borrelia burgdorferi* Sensu Stricto in South America? First Molecular Evidence of Its Presence in Colombia

**DOI:** 10.3390/tropicalmed7120428

**Published:** 2022-12-11

**Authors:** Lorys Y. Mancilla-Agrono, Lizeth F. Banguero-Micolta, Paula A. Ossa-López, Héctor E. Ramírez-Chaves, Gabriel J. Castaño-Villa, Fredy A. Rivera-Páez

**Affiliations:** 1 Programa de Biología, Facultad de Ciencias Exactas y Naturales, Universidad de Caldas, Calle 65 No. 26-10, Manizales 170004, Colombia; 2 Grupo de Investigación en Genética, Biodiversidad y Manejo de Ecosistemas (GEBIOME), Departamento de Ciencias Biológicas, Facultad de Ciencias Exactas y Naturales, Universidad de Caldas, Calle 65 No. 26-10, Manizales 170004, Colombia; 3 Facultad de Ciencias Exactas y Naturales, Universidad de Caldas, Calle 65 No. 26-10, Manizales 170004, Colombia; 4 Centro de Museos, Museo de Historia Natural, Universidad de Caldas, Calle 58 No. 21-50, Manizales 170004, Colombia; 5 Grupo de Investigación en Genética, Biodiversidad y Manejo de Ecosistemas (GEBIOME), Departamento de Desarrollo Rural y Recursos Naturales, Facultad de Ciencias Agropecuarias, Universidad de Caldas, Calle 65 No. 30-65, Manizales 17004, Colombia

**Keywords:** Lyme disease, Lyme borreliosis, emerging, amplifying host, reservoir

## Abstract

The genus *Borrelia* encompasses spirochetal species that are part of three well-defined groups. Two of these groups contain pathogens that affect humans: the group causing Lyme disease (LDG) and the relapsing fever group (RFG). Lyme disease is caused by *Borrelia burgdorferi* s.l., which is distributed in the Northern Hemisphere, and relapsing fevers are caused by *Borrelia* spp., which are found in temperate and tropical countries and are an emerging but neglected pathogens. In some departments of Colombia, there are records of the presence of *Borrelia* sp. in humans and bats. However, little is known about the impact and circulation of *Borrelia* spp. in the country, especially in wildlife, which can act as a reservoir and/or amplifying host. In this context, the objective of our research was to detect and identify the *Borrelia* species present in wild mammals in the departments of Caldas and Risaralda in Colombia. For morphological detection, blood smears and organ imprints were performed, and molecular identification was carried out through a nested PCR directed on the flagellin B (*flaB*) gene. A total of 105 mammals belonging to three orders (Chiroptera, Didelphimorphia and Rodentia) were analyzed, of which 15.24% (*n* = 16) were positive for *Borrelia*. Molecularly, the presence of *Borrelia burgdorferi* s.s. in lung tissues of *Thomasomys aureus* and blood of *Mus musculus* (Rodentia) was detected, with 99.64 and 100% identity, respectively. *Borrelia* sp. genospecies from a clade branch of a bat-associated LDG sister group were identified in seven individuals of bat species, such as *Artibeus lituratus*, *Carollia brevicauda*, *Sturnira erythromos*, and *Glossophaga soricina*. Furthermore, two *Borrelia* genospecies from the RFG in seven individuals of bats (*A. lituratus*, *Artibeus jamaicensis*, *Platyrrhinus helleri*, *Mesophylla macconnelli*, *Rhynchonycteris naso*) and rodents (*Coendou rufescens*, *Microryzomys altissimus*) were documented. Additionally, the presence of a spirochete was detected by microscopy in the liver of a *Sturnira erythromos* bat specimen. These results contain the first molecular evidence of the presence of *B. burgdorferi* s.s. in South America, which merits the need for comprehensive studies involving arthropods and vertebrates (including humans) in other departments of Colombia, as well as neighboring countries, to understand the current status of the circulation of *Borrelia* spp. in South America.

## 1. Introduction

The genus *Borrelia* (Spirochaetales: Borreliaceae) comprises about 43 recognized species of gram-negative motile spirochetes, ranging in length from 10–40 µm and 0.2–0.5 µm in diameter [1,2,3,4]. *Borrelia* are obligate vector-borne parasites that infect a wide diversity of vertebrate hosts, such as birds, reptiles, and mammals, including human as an accidental host [5,6,7,8]. Since the description of the genus *Borrelia* in 1907 [9], the number of described species and strains is increasing, grouping them into three well-defined phylogenetic groups: the Lyme disease or Lyme borreliosis group (LDG), the relapsing fever group (RFG), and the *Borrelia* associated with reptiles and monotremes group (RMG) [5,10].

Lyme disease (LD) is a multisystemic anthropozoonosis caused by the *Borrelia burgdorferi* s.l. complex, consisting of 20 recognized species, and with higher incidence in North America, Asia, and Europe [11,12,13,14,15]. Within *B. burgdorferi* s.l., there are six species of public health importance; however, *B. burgdorferi* s.s., *Borrelia garinii*, and *Borrelia afzelii* are the main etiological agents of LD in humans [15,16,17,18]. Vectors involved in LD transmission are hard ticks from the genus *Ixodes* (Ixodidae), in particular, *Ixodes ricinus*, *I. pacificus*, and *I. scapularis* [15,19]. Generally, the reservoirs are small rodents and birds, in which infection is asymptomatic [14,19]; in contrast, in humans and domestic animals such as dogs and cattle, a pathological syndrome can be triggered [14]. The most common clinical manifestation of the disease is erythema migrans [19,20,21]; however, when LD is not treated, spirochetes can colonize different tissues, leading to multi-organ clinical manifestations such as arthritis and cardiac conditions [22,23,24].

Similarly, relapsing fevers (RF) are infectious, neglected, and emerging diseases in the Americas and in some African countries, caused by 21 recognized species [4,25,26]. Vectors involved in the transmission cycle of relapsing fevers are mainly soft ticks of the genus *Ornithodoros* (Argasidae), hard ticks of the genera *Amblyomma*, *Dermacentor*, *Ixodes*, and *Rhipicephalus*, the human body louse or clothing louse *Pediculus humanus humanus*, the latter vector of *Borrelia recurrentis* [4,5,27,28,29,30,31,32]. *Borrelia* of the RFG exhibit transovarial and transstadial transmission in ticks, highlighting the importance of these vectors in the enzootic and epizootic cycles of the disease [4,29,32]. In addition, hosts and reservoirs of *Borrelia* of the RFG include rodents, opossums, and bats [30,32]; except for *Borrelia duttoni*, which is only known in humans as natural hosts [27]. Regarding the symptomatology of the disease, patients present episodes of high fever spaced by afebrile periods [16,30,33].

In recent decades, several tick-borne pathogens have been documented worldwide, including Lyme disease and relapsing fevers [34,35,36]. In recent years, several *Borrelia* genospecies have been reported using molecular tools in South America in both hosts and their vectors [37,38,39,40,41,42]. In Colombia, there are reports of the possible presence of *Borrelia* in humans since 1906, when *Borrelia*-like forms were first recorded in the country through blood smears of febrile patients in the Andean Region of the departments of Cundinamarca and Boyacá [43,44]; likewise, in 1907, a symptomatology compatible to RF was reported in the city of Manizales, Department of Caldas [45]. In addition, *Borrelia* spp. infection has been reported in humans in the Andean, Caribbean, and Pacific regions of the departments of Antioquia, Chocó, Córdoba, and Valle del Cauca [46,47,48,49,50], as well as in domestic animals in the departments of Antioquia and Cauca [51,52]. In Colombian wildlife, the presence of spirochetes has been reported in bats roosting at the Macaregua cave in the Department of Santander [40,53] and in the Department of Córdoba [54]; however, none of these reports correspond to *B. burgdorferi* s.s. Using metagenomic analysis, Carvajal-Agudelo et al. (2022) [55] reported the presence of *Borrelia* in bats and in *Ornithodoros hasei* soft ticks in the Department of Arauca.

Considering that *Borrelia* infections are usually subclinical, investigations of *Borrelia* spp. in wild mammals in Colombia are scarce, and that the impact and circulation of *Borrelia* spp. in the country is not well-known, the objective of the research was to determine the *Borrelia* species present in wild mammals and their associations with their hosts in the Andean Region of the Department of Caldas, Colombia.

## 2. Materials and Methods

### 2.1. Study Area

**Figure 1:**
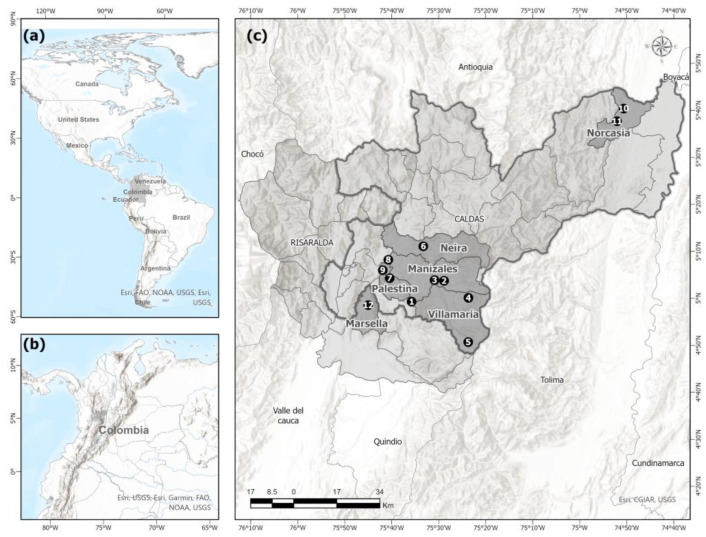
Study area. (**a**) American Continent, (**b**) Colombia, (**c**) Departments of Caldas and Risaralda, Colombia. Sampled localities in the municipalities of Manizales, Villamaría, Neira, Palestina, Norcasia, and Marsella. (1) Centro Nacional de Investigaciones de Café (CENICAFÉ). (2) Ecoparque Los Yarumos. (3) Jardín Botánico de la Universidad de Caldas. (4) Bosques de la CHEC. (5) Sector Santa Bárbara, finca El Edén. (6) Entrada Cementos Caldas. (7) Granja Montelindo de la Universidad de Caldas. (8) Vereda El Retiro. (9) Kilometro 35, Escuela. (10) Vereda Las Delicias, sector La Punta. (11) Vereda La Estrella, Finca El Encanto. (12) Río Cauca, túnel La Mica. Map created in ArcGis Pro 2.8.

The study was conducted in six municipalities of the Andean and inter-Andean valleys of Colombia (Figure 1): Manizales, Neira, Norcasia, Palestina, and Villamaría in the Department of Caldas and Marsella in the Department of Risaralda. The sampling sites were located in the basins of the Magdalena and Cauca Rivers, on both slopes of the Central Cordillera. This zone has an elevational range between 214 m and 5750 m, and the temperature varies according to the elevation [56,57].

### 2.2. Capture and Sampling of Mammals

Mammal capture was carried out using between two and six mist nets, 50–60 Sherman, and 10–12 Tomahawk traps per sampling site, using directed sampling without standardized efforts. One rodent (*Coendou rufescens*) was found dead in the study area. Each locality was sampled once for three to four days between March 2021 and April 2022. The Sherman and Tomahawk traps were installed on the first day of sampling between 10:00 and 12:00 h and were removed on the last sampling day between 6:00 and 8:00 h. The mist nets were installed and opened between 17:30 and 20:00 h. A capillary blood sample for blood smear was taken from each of the captured animals. To corroborate taxonomic identification, individuals were collected and euthanized following animal care recommendations [58,59]. Identification of the collected specimens was based on taxonomic keys [60,61] and subsequently deposited in the Mammal Collection of the Museum of Natural History of the University of Caldas (MHN-UCa). Wild mammal capture and collection were conducted with the approval of the Comité de Bioética de la Facultad de Ciencias Exactas y Naturales of the Universidad de Caldas (20 September 2019).

### 2.3. Morphological and Molecular Detection of Borrelia

To morphologically detect spirochetes, fine-drop blood smears (four plates per individual) and organ impressions on plates (two for each organ) were performed. Blood samples were obtained by puncture in the brachial vein (in bats) or by making a small cut at the end of the tail (in non-flying mammals), as well as through cardiac puncture after euthanasia. The organs (kidneys, liver, lungs, heart, and in some cases, brain and/or marrow), were arranged individually in sterile Petri dishes and washed with phosphate buffered saline (PBS) [62]. Each organ was then taken separately, washed again with PBS, cut longitudinally or transversely, excess blood was removed with sterile WypAll towels and pressed several times on the slide, and when necessary, several cuts were made. Additionally, and individually, each tissue was stored in absolute ethanol at 4 °C for subsequent molecular analysis (in paired organs, such as kidneys and lungs, a sample was taken from each one). All the materials used in the manipulation of the organs (forceps, scissors, Petri dishes) were washed with iodopovidone and distilled water and sterilized in a portable disinfection box (UV sterilization box 99% Obecilc I-lmh200317), including the WypAll towels. This process was performed each time a different organ was handled. Finally, the plates were fixed with absolute methanol for three minutes for blood smears and five minutes for organ impressions, then stained with 4% Giemsa solution for 40 min for blood and 45 min for organs. Each plate was reviewed using an OLYMPUS BX43 microscope and brightfield at the Laboratory of Molecular Biology of the Universidad de Caldas.

For the detection and molecular identification of *Borrelia* spp. extractions were performed for each individual and tissue, using the Wizard^®^ Genomic DNA Purification Kit (according to the standard protocol suggested by the manufacturer). In the DNA extraction, ultrapure water was used as a negative control, and in the PCR amplification, a reaction control was used in each reaction with *Borrelia anserina* and *Borrelia venezuelensis* as positive controls, which were donated by the Laboratório de Doenças Parasitárias under the direction of Dr. Marcelo Bahia Labruna of the Departamento de Medicina Veterinária Preventiva e Saúde Animal da Faculdade de Medicina Veterinária e Zootecnia, Universidade de São Paulo (USP-Brazil). Extracted DNA was subjected to nested PCR (nPCR) to amplify the flagellin B (*flaB*) gene: the first reaction mix was performed at a final volume of 20 µL with the external primer pair FLA LL 5′-ACATATTCAGATGCAGACAGAGAGGT-3′ and FLA RL 5′-ACATCATAGCCATTGCAGCAGACAGAGGT-3′, which amplified a 664 bp fragment. The mixture for the nested reaction was made at a final volume of 30 µL with the primers FLA LS 5′-AACAGCTGAAGAGAGCTTGGAATG-3′ and FLA RS 5′-CTTTGATCACTTATCATTCATTCTAATAGC-3′, which amplified a fragment of approximately 330 bp [63]. PCR products were evaluated by horizontal electrophoresis on 1% agarose gels stained with ethidium bromide, visualized on a GelDoc-It^®^ 2310 Image photodocumenter (UVP) (Thermo Fisher Scientific, Waltham, MA, USA), and sent to Macrogen (South Korea) for purification and sequencing.

### 2.4. Phylogenetic Analysis

Partial sequences of the *flaB* gene were evaluated and edited in Geneious Prime^®^ 2022.2.2 software [64]. Species identification and analysis considered similarity estimates with 50 public *Borrelia* sequences available in GenBank, including four sequences from Colombia and *Borrelia turcica* (associated with reptiles and monotremes) as outgroup. Sequence alignment was run in MAFFT [65] included in Geneious, and the best evolutionary model was selected in ModelFinder using AIC criterion [66]. A maximum likelihood (ML) phylogenetic analysis was performed in IQ-TREE [67], under the TIM3 + G4 + F model for 5000 ultrafast [68] bootstraps, as well as the Shimodaira–Hasegawa-like approximate likelihood ratio test (SH-like aLRT branch test) with 1000 replicates [69], included in the PhyloSuite platform [70], and the phylogenetic tree viewer FigTree v.1.4.3 [71] (Rambaut 2007) was used. Sequences obtained in this study were deposited in GenBank, and allelic matches were identified in *Borrelia* typing database at http://pubmlst.org/borrelia/ (accessed on 14 October 2022) [72]. Finally, genetic distances were estimated using the p-distance method with the MEGA 11 program [73]. Additionally, association networks were performed between *Borrelia* species and wild mammalian species, as well as between *Borrelia* species and the tissues evaluated; for this, R studio version 4.2.1 was used [74].

## 3. Results

A total of 105 mammals across 40 species, 10 families, and three orders (Didelphimorphia, Chiroptera, and Rodentia) were captured and analyzed (Table S1). A total of eight species of the order Chiroptera and four of the order Rodentia were found to be infected by *Borrelia* spp. with a prevalence of infection of 15.2% (*n* = 16). The families Phyllostomidae (Chiroptera) and Cricetidae (Rodentia) presented the highest number of infected species with seven and two, respectively (Table 1).

**Figure 2:**
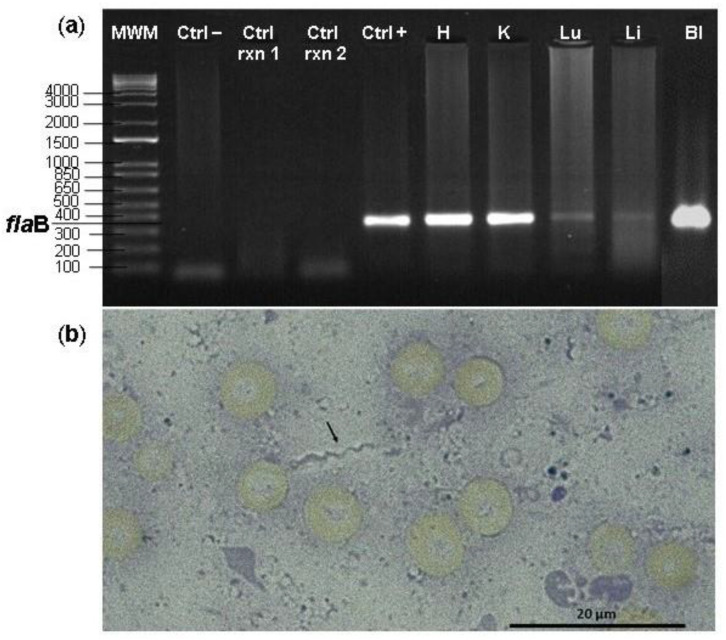
(**a**) Agarose gel showing the products of nested PCR targeting the flagellin B (*fla*B) gene from *Artibeus lituratus* tissues: (MWM): Molecular weight marker (1 Kb plus InvitrogenTM), (Ctrl −) negative control, (Ctrl rxn 1) first PCR reaction control, (Ctrl rxn 2) nested PCR reaction control, (Ctrl +) positive control *Borrelia anserina*, (H) heart, (K) kidney, (Lu) lung, (Li) liver, (Bl) blood. (**b**) Spirochete found in liver impression smear from a bat from *Sturnira erythromos* species.

A total of 470 tissue and organ samples (blood, liver, lung, heart, kidney, and brain) were examined. The prevalence of *Borrelia* spp. infection in samples was 5.1% (*n* = 24). In the liver, 25% (6/24) of the cases of infection were recorded, and in one bat specimen of *Artibeus lituratus* (Phyllostomidae), *Borrelia* sp. infection was present in all five tissues evaluated (except brain, which was not examined) (Table 1, Figure 2a). An individual of *Microryzomys altissimus* (Rodentia: Cricetidae) presented an exclusive infection in the brain with an identity of 99.61% with *Borrelia venezuelensis* [MG651650] from the RFG (Table 1). Additionally, by means of brightfield microscopy, a spirochete with an approximate length of 10.3 µm was detected in the liver of *Sturnira erythromos* (Figure 2b), while there was an LDG detection in the lung of the same individual.

Twenty-four (24) partial sequences of the *flaB* gene (~315 pb) were obtained and were deposited in GenBank with accession codes [OP491407-OP491408; OP480441-OP480462]. Seven of these sequences showed identity between 99.19%–99.64% with *B. venezuelensis* [MG651650] and *Borrelia turicatae* [MH632129] from the RFG. Two sequences showed an identity of 99.64% and 100% with *B. burgdorferi* s.s. [CP002228; AY374137] from the LDG, and 15 sequences showed an identity between 94.52% and 98.56% with one of the *Borrelia* genospecies [MT154618] found in Macaregua Cave in Colombia. Furthermore, four allelic variants were found in the *Borrelia* typing database, one for RFG and three for LDG (Table 1).

**Figure 3:**
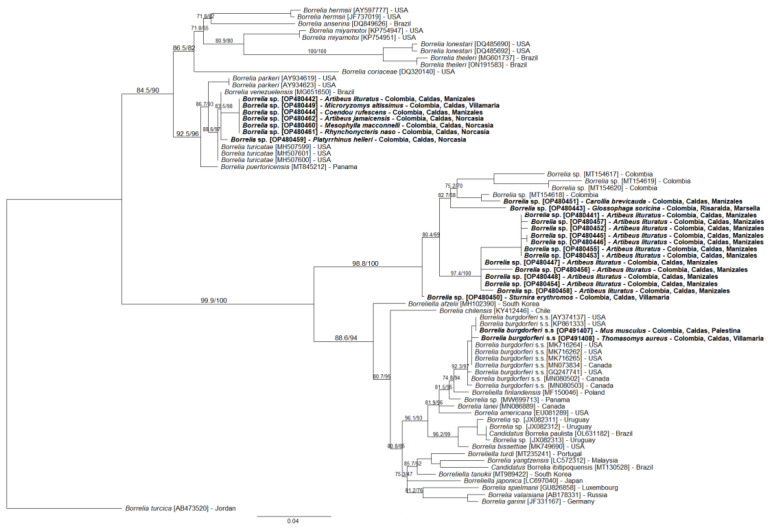
Phylogenetic tree of the partial sequences of the *flaB* gene of *Borrelia* species in the present study (in bold) and of the sequences in GenBank (accession numbers in parentheses), using the maximum likelihood (ML) method and the GTR + G4 + F model. Numbers at nodes are selected branch support analyses; from left to right: ultrafast bootstrap values, and Shimodaira–Hasegawa-like approximate likelihood ratio test (SH-like aLRT). The *Borrelia turcica* sequence was used as an outgroup.

The phylogenetic reconstruction obtained by ML using *flaB* sequences showed that two of the sequences of this study form a monophyletic clade with *B. burgdorferi* s.s. with a statistical support greater than 92% and a group with the sequences of the same species that were reported in the USA and Canada, with divergences between 0–1.1% (Figure 3; Table S2). Another monophyletic group was formed by 15 sequences of *Borrelia* sp. genospecies isolated from bats in Colombia (statistical support of 98.8%), which branches as a sister group of the LDG borreliae and are related to the sequences recorded in Macaregua Cave, Colombia (Figure 3; Table S2). Finally, the remaining seven sequences had genetic distances between them of 0–0.4%, are associated with the RFG, and are more closely related to *B. venezuelensis* (Brazil) with a statistical support of 83.5% in the ML tree and with genetic distances between 0–1.1% (Figure 3; Table S2).

**Figure 4:**
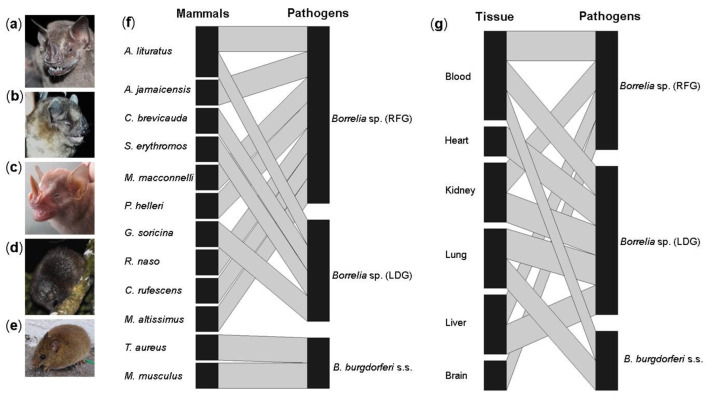
Association networks: (**a**) *Artibeus lituratus*, (**b**) *Sturnira erythromos*, (**c**) *Platyrrhinus helleri*, (**d**) *Coendou rufescens*, (**e**) *Microryzomys altissimus*; (**f**) associations between wild mammals and *Borrelia* species in the department of Caldas; (**g**) associations between wild mammal tissues and the *Borrelia* species found.

Finally, 13 associations between *Borrelia* and wild mammals were reported, involving *Borrelia* genospecies and 12 mammal species. *Borrelia* from the RFG presented the highest number of associations with several species of bats and rodents. In contrast, *Borrelia* genospecies branching as a sister group of the LDG were associated only with bats, and *B. burgdorferi* s.s. found in this study was associated with two rodent species: *Thomasomys aureus* (Rodentia: Cricetidae) and *Mus musculus* (Rodentia: Muridae) (Figure 4). Overall, blood was the tissue with the highest richness of *Borrelia* genospecies reported in this study (Figure 4g).

## 4. Discussion and Conclusions

Our results represent the first molecular evidence of the presence of *B. burgdorferi* s.s. in South America, a species that was considered absent in this region of the continent, where the presence of its known vectors is not recorded [16,75]. Nevertheless, the results obtained do not rule out the possibility that other ectoparasite species may be acting as vectors of these spirochetes, as recorded by other authors [38,76,77]. For South America, several Lyme disease group genospecies have previously been documented in both wild mammals and ticks [8,38,42,78,79,80]. In countries such as Brazil, the Baggio–Yoshinari syndrome has occurred, which is clinically similar to Lyme disease [81]; however, the causative agent could not be confirmed to be *B. burgdorferi* s.s. [82].

The ML reconstruction is congruent with previous reported phylogenetic inferences [37,40,52,80,83]. In particular, the *Borrelia* sequences recorded from bats in the present study are closely related to genospecies isolated from *Carollia perspicillata* in the Department of Santander, Colombia [40]. This relationship may indicate that the genospecies reported in our study and those recorded by Muñoz-Leal et al. (2021) [40] and Jorge et al. (2022) [80] are *Borrelia* restricted to bats, but further studies are needed to help support this hypothesis. 

Furthermore, the presence of *B. burgdorferi* s.s. in rodents (i.e., *T. aureus* and *M. musculus*) indicates their potential role as reservoirs for Lyme disease in the country, as has been observed in other rodents in Eurasia and North America [84,85]. Cricetid rodents (e.g., *Peromyscus leucopus*) have been evidenced to be competent reservoirs for *B. burgdorferi* s.l. in North America [84,85,86,87] and South America [8,42]. A high prevalence of infection by *B. burgdorferi* s.l. and other *Borrelia* species has been reported in other rodents such as murids [88,89]. Muridae have acquired great importance in zoonotic diseases, since their populations usually reach a large number of individuals that can act as hosts of ectoparasites and reservoirs of pathogens [89]. Similarly, some murids have colonized urban or rural environments, where they have close contact with humans and domestic animals, increasing the risk of infection [89]. 

The presence of *Borrelia* in the brain of *M. altissimus* coincides with the neurotropic characteristic of several *Borrelia* species [90,91], as is the case with *Treponema pallidum* [91]. *Borrelia* species are thought to infect the brain to evade the host immune response and may occasionally invade the blood to facilitate bacterial transmission [92]. This is likely the case for *B. duttonii* and *B. turicatae*, which have detected in the brains of rodents [91,92,93], and *B. miyamotoi* and *B. burgdorferi*, which were detected in the brain of rodents and a shrew (*Sorex maritimensis*) [94]. Due to the complexity of brain extraction, this organ was studied only in one specimen; therefore, we recommend including the brain in Lyme disease studies, as the ability of an infected host to potentially serve as a reservoir could be underestimated [95]. Additionally, the high prevalence of infection in the liver (25%) can be explained through one of the functions of this organ, which by filtering the blood, is likely to be one of the first tissues to be infected when spirochetes leave the circulatory system [94]. Furthermore, species of the *B. burgdorferi* complex have been shown to use the liver as a refuge to evade the immune response in long-term infections [96,97,98].

Finally, this research provides molecular evidence of the presence of *B. burgdorferi* s.s. in South America. Likewise, new associations between mammals and *Borrelia* sp. are presented for the American continent. These findings suggest that the presence of species of the genus *Borrelia* could be widely distributed in the wildlife of Colombia. However, complementary studies should be carried out to determine the real status of *Borrelia* spp. in South America and to establish the role played by wild mammals in the maintenance of infections by these spirochetes, as well as their participation in the enzootic and zoonotic cycles of *Borrelia* sp.

## Figures and Tables

**Table 1:** Mammals infected with *Borrelia* sp. in the departments of Caldas and Risaralda, Colombia. MHN-UCa-M: mammal collection, Museo de Historia Natural, Universidad de Caldas.

Municipality	Locality	Host Order	Host Family	Voucher Number MHN-UCa	Host’s Scientific Name	Infected Tissue	Accession Number	Closest Gen Bank Identity (Gene: Accession Number)	*Borrelia* Typing Database (Locus/Allele)
Manizales	Jardín Botánico, Universidad de Caldas	Chiroptera	Phyllostomidae	M 3826	*Artibeus lituratus*	Blood	OP480442	99.64% *Borrelia venezuelensis* [*fla*B: MG651650]	BORR00725 (*fla*B)/53
				M 3821	*Artibeus lituratus*	Blood	OP480441	95.17%–96.24% *Borrelia* sp. Macaregua cave [*fla*B: MT154618]	/37
						Heart	OP480445		/37
						Kidney	OP480446		/37
						Lung	OP480447		/37
						Liver	OP480448		/37
				M 3820	*Artibeus lituratus*	Heart	OP480454	95.75%–96.61% *Borrelia* sp. Macaregua cave [*fla*B: MT154618]	/37
						Kidney	OP480452		/37
						Lung	OP480453		/37
				M 3822	*Artibeus lituratus*	Heart	OP480457	94.52%–95.21% *Borrelia* sp. Macaregua cave [*fla*B: MT154618]	/37
						Kidney	OP480455		/37
						Liver	OP480456		/37
				M 3825	*Artibeus lituratus*	Kidney	OP480458	96.18% *Borrelia* sp. Macaregua cave [*fla*B: MT154618]	/37
Norcasia	Vereda Las Delicias, Sector La Punta, Río Manso			M 3987	*Artibeus jamaicensis*	Liver	OP480462	99.63% *Borrelia venezuelensis* [*fla*B: MG651650]	/53
Manizales	Jardín Botánico, Universidad de Caldas			M 3812	*Carollia brevicauda*	Lung	OP480451	98.56% *Borrelia* sp. Macaregua cave [*fla*B: MT154618]	/37
Villamaría	Bosques de la CHEC			M 3529	*Sturnira erythromos*	Lung	OP480450	96.32% *Borrelia* sp. Macaregua cave [*fla*B: MT154618]	/37
Norcasia	Vereda Las Delicias, Sector La Punta, Río Manso			M 3984	*Platyrrhinus helleri*	Kidney	OP480459	99.62% *Borrelia venezuelensis* [*fla*B: MG651650]	/53
				M 3981	*Mesophylla macconnelli*	Liver	OP480460	99.28% *Borrelia venezuelensis* [*fla*B: MG651650]	/53
Marsella *	Rio Cauca, túnel La Mica			M 3635	*Glossophaga soricina*	Blood	OP480443	95.19% *Borrelia* sp. Macaregua cave [*fla*B: MT154618]	/29
Norcasia	Vereda Las Delicias, Sector La Punta, Río Manso		Emballonuridae	M 3970	*Rhynchonycteris naso*	Liver	OP480461	99.65% *Borrelia venezuelensis* [*fla*B: MG651650]	/53
Manizales	Vía Viveros, Ecoparque Los Yarumos	Rodentia	Erethizontidae	M 3423	*Coendou rufescens*	Liver	OP480444	99.19% *Borrelia turicatae* [*fla*B: MH632129]	/53
Villamaría	Sector Santa Bárbara, Finca El Edén		Cricetidae	M 3613	*Microryzomys altissimus*	Brain	OP480449	99.61% *Borrelia venezuelensis* [*fla*B: MG651650]	/53
				M 3616	*Thomasomys aureus*	Lung	OP491408	99.64% *Borrelia burgdorferi* [complete genome: CP002228; *fla*B: AY374137]	/62
			Muridae	M 3605	*Mus musculus*	Blood	OP491407	100% *Borrelia burgdorferi* [complete genome: CP002228; *fla*B: AY374137]	/62

* Mammals infected in the departments of Risaralda, Colombia.

## Data Availability

The data presented in this study are contained within the article.

## Supplementary Materials

The following supporting information can be downloaded at https://www.mdpi.com/article/10.3390/tropicalmed7120428/s1: Table S1: Wild mammals sampled (Departments of Caldas and Risaralda). Individuals that were positive for *Borrelia* are highlighted in bold.—indicates that the information is not available; Table S2: Intraspecific and interspecific distances based on the p-distance method for the flagellin gene fragment (*fla*B) with the MEGA v. 11 program. In bold are the sequences obtained in this study and the GenBank accession numbers are provided in parentheses.
